# A proof-of-concept investigation into ketamine as a pharmacological treatment for alcohol dependence: study protocol for a randomised controlled trial

**DOI:** 10.1186/s13063-017-1895-6

**Published:** 2017-04-04

**Authors:** Amy McAndrew, Will Lawn, Tobias Stevens, Lilla Porffy, Brigitta Brandner, Celia J. A. Morgan

**Affiliations:** 1 Psychopharmacology and Addiction Research Centre (PARC), College of Life and Environmental Science, University of Exeter, Washington Singer Building, Perry Road, Exeter, EX4 4QG UK; 2 Clinical Psychopharmacology Unit, University College London, 1-19 Torrington Place, London, WC1E 7HB UK; 3 Anaesthetics Department, Podium 3, maple Link corridor, Unviersity College Hospital, 235 Euston Road, London, NW1 2BU UK

**Keywords:** Alcoholism, Ketamine, Relapse, Depression

## Abstract

**Background:**

Worldwide, alcohol abuse is a burgeoning problem. Abstinence is key to allow recovery of physical and mental health as well as quality of life, but treatment for alcohol dependence is associated with high relapse rates. Preliminary data have suggested that a combined repeated ketamine and psychological therapy programme may be effective in reducing relapse in severe alcohol use disorder. This non-commercial proof-of-concept trial is aimed at making a preliminary assessment of the effectiveness of this combined treatment in this patient group.

**Methods/design:**

This is a phase II, randomised, double-blind, placebo-controlled, parallel-group clinical trial taking place in two sites in the UK: the South West of England and London. Ninety-six recently detoxified alcoholics, with comorbid depressive symptoms, will be randomised to one of four treatment arms. Patients will receive either three sessions of ketamine (0.8 mg/kg administered intravenously (IV) over 40 minutes) or placebo (50 ml saline 0.9% IV over 40 minutes) plus either seven sessions of manualised psychological therapy or an alcohol education control. Patients will be assessed at 3 and 6 months on a range of psychological and biological variables. The primary endpoints are (1) relapse rates at 6 months and (2) percentage days abstinent at 6 months. Secondary endpoints include 3 and 6 month percentage days abstinence, tolerability (indicated by dropout), adverse events, depressive symptoms, craving and quality of life.

**Discussion:**

This study will provide important information on a new combined psychological and pharmacological intervention aimed at reducing relapse rates in alcoholics. The findings would have broad application given the worldwide prevalence of alcoholism and its associated medical, psychological and social problems.

**Trial registration:**

ClinicalTrials.gov, NCT02649231. Registered on 5 January 2016.

**Electronic supplementary material:**

The online version of this article (doi:10.1186/s13063-017-1895-6) contains supplementary material, which is available to authorized users.

## Background

Alcohol misuse globally accounts for roughly 4% of all deaths and 5% of the burden of disease [1]. In the UK alone, nearly 9% of men and 4% of women today meet criteria for alcohol dependence — in all approximately 3.9 million British adults [2]. In the UK, 22,000 people die annually because of alcohol misuse, which can produce severe and enduring physical and neurological problems. Costs to the National Health Service (NHS) from alcohol abuse are estimated at £2.7 billion annually [1]. Abstinence is pivotal in order to tackle problems associated with alcohol dependence, but alcohol dependence is associated with high relapse rates.

Pilot work conducted in the 1980s found unprecedented reductions in relapse rates in alcoholism following ketamine treatment. Three weekly doses (2.5 mg/kg bolus IM) of ketamine were given alongside sessions of psychotherapy before and after the administration of the drug to 111 recently detoxified alcohol-dependent patients. One year later, 66% of the patients in this group were still abstinent. In comparison, out of a control group of 100 similar recently detoxified alcohol-dependent patients, 24% were abstinent after 1 year [3]. A 40% reduction in relapse rate is much greater than anything previously observed with any other relapse prevention method in alcohol dependence. However, in the latter study patients could freely choose which treatment condition they wanted to experience, and so the experiment was neither blinded nor a randomised controlled trial. Nevertheless, these preliminary findings have been supported by case studies of the successful use of ketamine, alongside transpersonal therapy, in the treatment of alcoholism, where 15 patients had 70% abstinence rates at 1 year [4].

At the time of the pilot work in alcohol dependence in Russia, any potential biological mechanism was unclear. However, the remarkable recent success of isolated doses of ketamine in the treatment of depression [5, 6] suggests that a similar biological mechanism could be in operation in this observed reduction of relapse in alcoholism following ketamine. The proposed biological mechanism behind the anti-depressant effects of ketamine is increased synaptogenesis, and recent empirical evidence suggests that blocking synaptogenesis also blocks ketamine-induced reductions in alcohol consumption [7]. We are proposing that ketamine may reduce relapse in alcoholism via stimulating the growth of neurons and synapses and enhancing the uptake of psychological therapy.

Recent theoretical accounts have proposed a role for impaired neurogenesis and synaptogenesis in addiction [8, 9]. These accounts are supported by pre-clinical studies that suggest that these processes are blocked following administration of addictive substances, in particular alcohol (see, e.g. [10–15]). Changes in potential indices of neurogenesis, such as serum brain-derived neurotrophic factor (BDNF), are observed in alcoholics [16], with levels increasing with their sobriety [17]. Reducing the numbers of newborn neurons and synapses has been suggested as a means of making the addicted brain less able to learn about non-drug rewards and unable to project into the future [8].

Psychosocial therapies are currently the mainstays of treatment for alcohol dependence but are of limited effectiveness, which may partly be because they depend on the brain’s capacity for neuroplastic change [18]. Psychological therapies in alcoholism are also less effective in individuals with cognitive impairment [19]. Undergoing psychological therapy, which involves learning and thinking in different ways, during the period of neurogenesis and synaptogenesis after an acute dose of ketamine [20] may facilitate its uptake, as the brain is thus primed to make new connections and learn new information. Increased synaptogenesis has been linked with improved memory (see, e.g. [21, 22]). Therefore, ketamine may enhance the uptake of psychological therapy on the day following an acute dose by making the brain more receptive to new learning.

In order to examine whether enhancing the uptake of therapy is the mechanism by which ketamine is effective in alcoholism, we have included a ketamine with ‘no therapy’ (i.e. alcohol education) arm in the trial. These data will have considerable implications not only for alcohol dependence, but also depression, since patients with comorbid depressive symptoms are being recruited for this trial. Positive findings may suggest that a similar psychotherapy plus ketamine approach may enhance the effectiveness of ketamine treatment in patients with depressive symptoms.

The research question is whether ketamine can reduce relapse in alcohol dependence. To address this question, we are carrying out a phase II, multi-site, randomised, double-blind, placebo-controlled, parallel-group clinical trial investigating the impact of a novel brief pharmacological and psychological intervention aimed at reducing relapse rates in recently detoxified alcohol-dependent individuals with depressive symptoms. We predict that ketamine compared to placebo will reduce relapse at 6 months. Additionally we hypothesise that ketamine will have a synergistic effect with psychological therapy, reducing relapse rates at 6 months (increasing the percentage of days abstinent) as compared to ketamine plus alcohol education control.

## Methods/design

### Overview of study design

This study is a non-commercial, randomised, double-blind, parallel-group clinical trial with four treatment arms: (1) ketamine and psychological therapy, (2) ketamine and alcohol education, (3) saline and psychological therapy and (4) saline and alcohol education. Eligible participants will be recruited from two study sites in the UK, London and the South West of England.

### Eligibility criteria

All prospective patients will be assessed to determine eligibility by the study team according to the inclusion and exclusion criteria outlined below. Every prospective patient will be informed about the trial design and protocol by the study team and will be encouraged to discuss suitability with a non-study member.

#### Inclusion criteria

The inclusion criteria are as follows:

- 18 to 60 years old.
- Meet either (1) Diagnostic and Statistical Manual of Mental Disorders (DSM)-5 criteria for severe alcohol use disorder or (2) DSM-IV criteria for severe alcohol dependence within the last 12 months.
- Currently abstinent from alcohol (breathalyser blood alcohol concentration (BAC) level 0.00) and negative urine drug screen (participants testing positive for tetrahydrocannabinol (THC) who do not have a history or current cannabis dependency may be included). THC dependency will be assessed by general practitioner (GP) correspondence as well as self-disclosure of seeking help for a cannabis dependency. THC use will be assessed using urine drug tests at each trial visit as well as during drug histories administered at the screening visit and at visits 8, 9 and 10.
- Minimum of mild depression (>14 on Beck Depression Inventory-II).
- Capacity to give informed consent as defined by Good Clinical Practice (GCP) guidelines.
- Willing and able to wear Secure Continuous Remote Alcohol Monitor (SCRAM-X) bracelet for 6 months.
- Females of childbearing potential and males must be willing to use an effective method of contraception (hormonal or barrier method of birth control; true abstinence) from the time consent is signed until 6 weeks after treatment discontinuation and inform the trial if pregnancy occurs.
- Females of childbearing potential must have a negative pregnancy test within 7 days prior to being registered for trial treatment and on the day of first treatment.

#### Exclusion criteria

The exclusion criteria are as follows:

- Currently taking any other relapse prevention medication or anti-depressants
- Uncontrolled hypertension (systolic 140 mm Hg or greater and diastolic 90 mm Hg or greater)
- Body mass index (BMI) outside normal limits (<16 and >35)
- History of psychosis, or psychosis in a first-degree relative, as identified by DSM-5 or DSM-IV Structured Clinical Interview (SCID); co-morbid psychiatric diagnosis excluding depression, identified via self-report or notified by their medical professional
- Previous or current diagnosis of substance dependence/severe substance misuse disorder
- History of neuropsychological difficulties
- One or more confirmed seizures
- Currently taking daily prescribed medication contraindicated in the Summary of Product Characteristics (SPC)
- Liver function tests greater than three times normal levels
- Where there are special warnings or precautions for use according to the SPC where the risk benefit ratio is not in favour of giving ketamine with assessment made by physical examination by medically qualified trial personnel, self-report or inspection of the medical notes:
  - ◦ Acute intermittent porphyria
  - ◦ Dehydration or hypovolaemia
  - ◦ Hyperthyroidism, or patients receiving thyroid replacement
  - ◦ Pulmonary or upper respiratory tract infection
  - ◦ Severe coronary artery disease, cerebrovascular accident or cerebral trauma
  - ◦ Diabetes
  - ◦ Known glaucoma or globe injuries
  - ◦ Cirrhosis
  - ◦ Epilepsy
  - ◦ Neurological condition/brain damage
  - ◦ Intracranial mass lesions, presence of head injury or hydrocephalus
- Suicidal ideation
- Not willing to use effective contraception or (females) take pregnancy test
- Allergic reaction to ketamine
- More than ten previous detoxifications from alcohol
- Pregnant or breastfeeding
- Allergies to excipients of the investigational medicinal product (IMP) or placebo
- Use of another IMP that is likely to interfere with the study medication within 3 months of study enrolment

### Randomisation, blinding and unblinding

Simple randomisation will be used to allocate patients using a 1:1:1:1 ratio for the four respective study arms. Randomisation will be performed by a specialist company (www.sealedenvelope.com) who will hold a randomisation list and provide 24/7 Internet access for unblinding services. Early dropouts (during screening and up until randomisation) will be replaced. Once patients have been randomised, they will not be replaced regardless of compliance or dropout status and will be analysed as part of the intention-to-treat analysis. Randomisation will be performed by delegated medical staff who are satisfied with patient eligibility to participate. Two emails will be sent on randomisation, one to delegated staff preparing the IMP detailing whether the IMP or placebo should be provided for infusion, and another to the psychologists determining whether therapy or education should be undertaken.

Upon randomisation, patients will be provided with a study-specific patient card which will have details regarding the study title, IMP details, patient ID and contact details of the out-of-hours contact in cases of emergency. The study code should only be broken for valid medical or safety reasons, e.g. in the case of a severe adverse event where it is necessary for the investigator or treating health care professional to know which treatment the patient is receiving before the patient can be treated. Subject always to clinical need, where possible, members of the research team will remain blinded. The code breaks for the trial are held at www.sealedenvelope.com. Authorised members of the research team will have administrative rights to the unblinding system, and a nominated individual will be available 24 hours a day.

### Trial structure

**Fig. 1:**
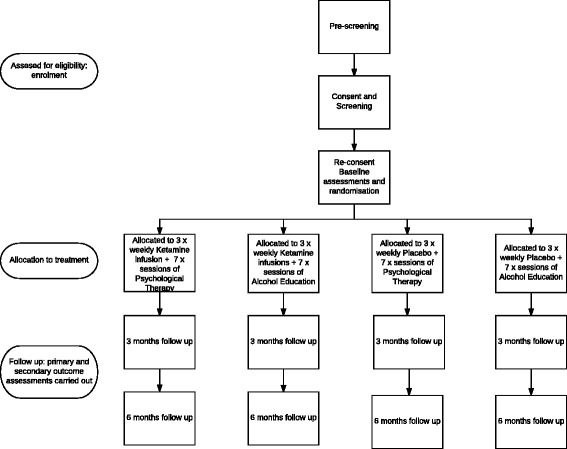
KARE trial design. Step 1: Participants are initially identified and go through pre-screening. Step 2: If eligible, participants are invited for a screening visit to further determine eligibility. Step 3: If eligible, participants are randomised into one of four treatment conditions: (1) ketamine + relapse prevention therapy, (2) ketamine + alcohol education, (3) placebo + relapse prevention therapy, (4) placebo + alcohol education. Treatment runs over 7 sessions (1 therapy/education per session, ketamine/placebo is administered in 3 sessions). Step 4: Participants are followed up at 12 weeks. Step 5: Participants are followed up at 24 weeks

**Fig. 2:**
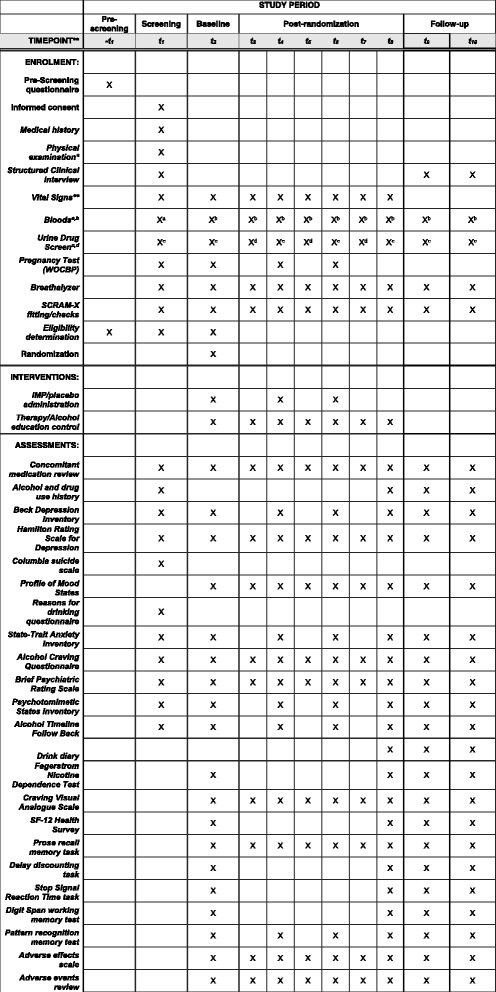
KARE trial SPIRIT checklist. This checklist contains all trial-specific assessments that will be run on each study visit. An ‘X’ denotes that this assessment will be run. * Physical examination: cardiovascular, respiratory, gastrointestinal and neurological assessment to a level of detail that would be expected for a patient due to receive anesthesia. ** Vital Signs: Resting pulse, pulse oximetry and blood pressure for safety monitoring. Vital signs will also be continuously monitored throughout the infusion until the participant has recovered. X^a^ Bloods: Full Blood Count (haemoglobin, white cell count, platelets, mean red cell volume); Liver Function Tests (Bilirubin, Alanine aminotransferase, Asparate aminotransferase, Total Protein, Alkaline phosphatase, Albumin, Globulin, gamma-glutamyl transpeptidase), Biochemistry (urea, sodium, potassium, glucose, calcium, thyroid stimulating hormone). X^b^ Bloods: Liver Function Tests (same as above); Brain-derived neurotrophic factor; ketamine. X^c^ Urine drug screen panel (methamphetamine, cocaine, tetrahydrocannabinol, benzodiazepines, tricyclic antidepressants, barbiturates, phencyclidine, amphetamines, morphine, methadone) including ketamine. X^d^ Urine drug screen panel (same as above) excluding ketamine

Patients involved in this trial will go through ten study visits, an overview of which can be seen in Fig. 1. Specific details about study activities that will be undertaken at each visit can be found in the Standard Protocol Items: Recommendations for Interventional Trials (SPIRIT) checklist (Additional file 1) and in Fig. 2.

### Pre-screening assessment

Patients identified as wanting to take part in this study will receive a telephone assessment by a member of the research team to discuss the trial and confirm initial eligibility using the inclusion and exclusion criteria outlined above. Where applicable, a screening visit will be booked for those who pass the initial eligibility and still express interest in participating.

### Screening visit

Formal screening will take place at one of the two study sites. Consent will be taken from the patient prior to any assessments at this visit. Following this, a variety of physical and psychological assessments will be undertaken, details of which can be found in the SPIRIT checklist, in order to further determine eligibility. Those still meeting criteria for inclusion at the end of this visit will be fitted with the SCRAM-X bracelet, and a date for the baseline visit will be arranged within 14 days of the screening date. In between study visits a delegated anaesthetist will determine whether patients are potentially eligible to be randomised into the trial.

### Intervention

#### Baseline assessments

At the start of the baseline visit, certain assessments will be undertaken to ensure eligibility is still reliable. Patient vital signs will be checked, and a concomitant medication review will be undertaken, along with a breathalyser reading and urine drug screen and, where applicable, a pregnancy test. The SCRAM-X bracelet will be checked. If the patient is happy and is eligible, the anaesthetist will randomise the patient into the trial, and baseline data will be collected from a variety of psychological assessments, details of which can be found in the SPIRIT checklist. A session of either relapse prevention-based psychological therapy or an alcohol education session will then be administered to the patient depending on treatment allocation. Subsequently, an infusion of either the IMP or placebo will be administered. Further details about all four treatment allocations are detailed below.

#### Relapse prevention-based psychological therapy

Across the initial baseline and subsequent six study visits, half of the patients will receive psychological therapy. This is a manualised therapy developed with alcohol specialist clinical psychologists. This manual is not available in published form. The aim of the seven sessions is to help to develop an enjoyable and meaningful life without alcohol. It focuses around two broad themes. The first theme relates to relapse prevention, i.e. skills and strategies to reduce the likelihood of relapse and maintain abstinence. The second theme focuses on promoting well-being and deals with issues such as purpose, meaning and life enjoyment, along with skills of coping with life’s day-to-day problems and stressors. Each session is designed to last one hour and contains one topic related to relapse prevention and another to promoting well-being. In between these two main themes a different relaxation or mindfulness exercise is introduced.

#### Alcohol education control

Half of the patients in this trial will receive seven sessions of alcohol education, educating patients about the risks of alcohol use and the effects of alcohol on the body. The focus will be on topics including the driving forces of addiction, the biological effects of alcohol, the way that drugs interact with the brain, alcohol’s relationship with other illnesses and ways to generally improve healthful living. The alcohol education sessions will have no psychological components relating to personal relapse prevention strategies or the promotion of personal well-being. Each session has been designed to last one hour to be comparable to the psychological therapy sessions. These education sessions should equate the amount of personal interaction each patient has between the two conditions (therapy and education), meaning interpersonal interaction should not be responsible for any differences on the research outcomes between these conditions.

#### IMP/saline placebo

Patients will receive three weekly intravenous infusions of ketamine (Ketalar®), 0.8 mg/kg made up to 50 ml with saline over 40 minutes, or three weekly infusions of placebo, 0.9% saline over 40 minutes. The ketamine/placebo will be prepared in a blinded fashion by pharmacy or delegated unblinded site staff and will be administered by the research anaesthetist at the clinical research facility who will remain blinded.

#### Subsequent study visits

Over the course of the trial, three visits will fall the day after the preceding infusion visits (visits 3, 5 and 7). These visits will importantly involve a session of relapse prevention-based therapy or alcohol education. Visit 8 delivers the last psychological therapy or alcohol education session in this trial. Visits 4 and 6 will be the two other visits in which there is an infusion of ketamine or placebo (as well as the baseline visit). Visits 9 and 10 are the corresponding 3- and 6-month follow-up visits. The SPIRIT checklist contains a complete list of all assessments that will be conducted at each study visit.

### Adverse event reporting

All adverse events will be recorded in the medical records and case report form (CRF) only following consent. If an investigator suspects that the patient’s disease has progressed faster due to the administration of the IMP, then he/she will record and report this as an unexpected adverse event. However, adverse events that are known to the IMP when used in its licenced indication will only be recorded in the medical notes, while serious adverse events will be recorded in the CRF and serious adverse events log. Clinically significant abnormalities in the results of objective tests (e.g. liver function, biochemistry) will also be recorded as adverse events. If the results are not expected as part of disease or IMP, they will also be recorded as unexpected. All adverse events will be recorded until 6 months post-treatment. All serious adverse events will be reported to the sponsor from IMP administration up to 30 days after last IMP administration.

### Recruitment and timeline

Recruitment of the 96 patients across the two study sites in England commenced in July 2016 and is expected to last 2 years. Our justification of this sample size is as follows: with *n* = 24 people in each drug arm we will be able to estimate a decrease in the relapse rate from 50% in patients given placebo to 25% (90% confidence interval: 3–47%) in patients given ketamine.

A decrease of 25% in relapse would be clinically important and lower than that observed in the preliminary work ([3]: 42%); it would clearly suggest that further investigation is warranted. Our confidence intervals are relatively broad, as this is a proof-of-concept study, and we wish to minimise the numbers of subjects involved.

Patients will be primarily recruited from Participant Identification Centres (PICs). These centres will be the substance misuse and addiction psychiatry services in Central, North West and South London as well as Devon, Dorset, Wiltshire, Somerset and Bristol. These centres will refer potential patients to the research team. If interested, PICs will provide the patient with a Research Ethics Committee (REC)-approved letter of invitation that contains details for the patient to contact the study team directly. The research team will then ensure that a copy of the Participant Information Sheet (PIS) is made available to the patient prior to any screening assessments. All patients will be included at both follow-up periods of 3 and 6 months. The actual timeline may be slightly different depending on patient eligibility.

A study website will also be set up to accompany the study, where patients can register their interest and the trial will be publicised through forums and social networking, relevant charities such as Foundation 66 and Alcohol Concern, newspaper, radio and TV, in advice centres and via word of mouth and face-to-face contact.

### Database management

Anonymised trial data will be stored in a bespoke electronic CRF hosted on sealedenvelope.com, prepared using their ‘Red Pill’ service. Trial-specific documents held by researchers will be stored securely with access restricted and limited to nominated research staff and in accordance with the Data Protection Act 1998, University College London (UCL) Information Security Policy and Trust Information Governance Policy. Electronic and questionnaire source data will be stored in a password-protected Microsoft Access database from where summary data will be transcribed into the eCRF. The Red Pill database will keep a record of all changes to and access of the database.

### Definition of endpoints and outcome measures

#### Primary outcomes

The primary outcomes are the following:

- Relapse rates at 6 months
- Percentage days abstinent at 6 months

#### Secondary outcomes

Secondary outcomes are as follows:

- Number of days of continuous abstinence at 3 months
- Percentage days abstinent at 3 months
- State mood
- Depression
- Anxiety
- Psychotic symptoms
- Cigarette smoking
- Craving
- Quality of life
- Episodic memory
- Delay discounting
- Response inhibition
- Working memory
- Hippocampal functioning
- Adverse effects

For the purposes of this trial, relapse is defined as 5 drinks (8.1 units of alcohol) or more in men and 4 drinks or more (6.5 units of alcohol) in women on a single occasion.

### Statistical methods

#### Primary outcome analysis

All analyses will be performed by a statistician blinded to the treatment allocations and will be performed using Stata v.13. The proportion of relapsed patients at the 6-month follow-up will be reported as a percentage for each arm, as well as for the ketamine arms combined and the psychotherapy arms combined. The proportion of relapsed participants at the 6-month follow-up will be expressed as a relative risk with a 95% confidence interval for the following comparisons: (1) all ketamine patients versus all non-ketamine patients; (2) ketamine and psychotherapy patients versus ketamine and education patients; and (3) ketamine and psychotherapy patients versus placebo and education patients. These analyses are purely exploratory and should be viewed with caution both in light of lack of power and multiple testing. Percentage of days abstinent at the 6-month follow-up will be reported descriptively in the same way by arm and combination of arms; additionally between-group mean difference and 95% confidence intervals will be reported for the comparisons set out above; again, these analyses should be regarded as exploratory. The primary analysis will be an intention-to-treat (ITT) complete case analysis including all randomised participants. A secondary analysis will be conducted including only those patients deemed to have completed to a sufficient degree their allocated interventions, that is, a per protocol analysis.

#### Secondary outcome analysis

There are 15 secondary outcomes; all of these outcomes will be reported descriptively on an ITT complete case basis. Additionally, the between-group differences and 95% confidence intervals will be reported on an exploratory basis for the primary comparison (all ketamine patients versus all non-ketamine patients). Also, as a feasibility outcome, attrition will be reported as a proportion (with 95% confidence interval) overall and by arm.

#### Sensitivity and other planned analyses

All patients will be in a state of detoxification at recruitment; hence, in the event of loss of follow-up, it is not appropriate to make an assumption that the patient had or had not relapsed at 6 months. To address the issue of missing data due to loss to follow-up, a sensitivity analysis will be performed using multiple imputation methods, and for relapse a 6-month follow-up only, making the assumption that all missing data were missing at random.

### Termination criteria

The end of the trial will be the last follow-up visit of the patient at 24 weeks post-baseline. All patients have the right to withdraw at any time. In addition the Chief Investigator (CI) may discontinue a patient from the study at any time it is considered necessary for any reason including:

- Ineligibility (either during the study or retrospective)
- Significant protocol deviation
- Significant non-compliance with treatment or study requirements
- An adverse event which requires discontinuation of the study medication or results in inability to continue or comply with study procedures
- Disease progression which requires discontinuation of the study medication or results in the inability to comply with study procedures
- Consent withdrawn, loss of capacity or detention under the Mental Health Act
- Pregnancy
- In the case of an overdose

However, all efforts will be made to maintain patients in the study where possible and to continue assessments as intended. If treatment is stopped for longer than one week in this study, then it will not be possible to resume it. The reason for participant withdrawal will be recorded in the CRF.

Every effort will be made to attempt to continue full protocol assessment from dropouts and/or patients who repeatedly violate the protocol on the basis of our planned ITT analysis. The trial may be stopped before completion for the following reasons: (1) if the CI and/or sponsor decide to suspend the trial pending safety review of an emergent issue or (2) if the CI and/or sponsor decide to stop the trial for safety, administrative or other reasons.

## Discussion

Ketamine has a very favourable safety profile. One concern with this therapy may be that ketamine can also be abused, but we have shown that higher subanaesthetic doses of ketamine, such as that proposed in this study, are not rewarding [23], and recently detoxified alcoholics given ketamine did not go on to abuse the drug [21, 24]. Also, alcohol craving did not increase following ketamine use in this group [24]. A clinical trial at Yale University in the USA using ketamine for the treatment of depression in alcoholism [25] reported the treatment to be well tolerated with no adverse events (Petrakis IL, personal communication). Further, there has been no evidence of subsequent drug abuse or any persisting problems when ketamine was administered to a large sample of psychologically prepared patients in a supportive research setting [26], and similar absences of subsequent drug abuse have been observed in smaller samples of depressed patients [27].

Benefits of no longer being dependent on alcohol for end users are considerable and wide-ranging. Physical health would improve, and risks of alcohol-related diseases such as cirrhosis of the liver would decrease. Benefits would also be observed in terms of end users’ mental health (depression, anxiety) and improved cognitive function, resulting in an overall increase in quality of life. Considering the high toll alcohol abuse has on the health system [27], benefits would accrue in a reduction in burden on the NHS and its workforce, in alcohol-related deaths and in other non-fatal acute harms. Economic benefits would not only be reflected in a reduction in burden on the NHS but also in more frequent and regular engagement in work activities and a reduction in crime. Importantly for end users, the brief nature of our pharmacological intervention will be less stigmatising than current treatments that require taking pharmacotherapies for prolonged periods of time.

## Trial status

This trial is currently recruiting patients at both study sites.

## Additional file

Additional file 1: KARE trial SPIRIT checklist detailing assessments made in the trial. (DOC 122 kb)

## Abbreviations

BMI: Body mass index
CI: Chief Investigator
CRF: Case report form
DSM: Diagnostic and Statistical Manual of Mental Disorders
IMP: Investigational medicinal product
ITT: Intention-to-treat
PIC: Participant Identification Centre
PIS: Participant Information Sheet
REC: Research Ethics Committee
SCRAM-X: Secure Continuous Remote Alcohol Monitor bracelet
SPC: Summary of Product Characteristics
WOCBP: Woman of child bearing potential
